# The association between 25-hydroxyvitamin D concentration, physical performance and frailty status in older adults

**DOI:** 10.1007/s00394-018-1634-0

**Published:** 2018-04-25

**Authors:** Anouk M. M. Vaes, Elske M. Brouwer-Brolsma, Nicole Toussaint, Margot de Regt, Michael Tieland, Luc J. C. van Loon, Lisette C. P. G. M. de Groot

**Affiliations:** 1 Division of Human Nutrition, Wageningen University, P.O. Box 8129, 6700 EV Wageningen, The Netherlands; 2 Top Institute Food and Nutrition, P.O. Box 557, 6700 AN Wageningen, The Netherlands; 3 Department of Human Biology and Movement Sciences, NUTRIM School for Nutrition and Translational Research in Metabolism, Maastricht University Medical Centre, P.O. Box 616, 6200 MD Maastricht, The Netherlands

**Keywords:** 25-Hydroxyvitamin D, Vitamin D, Muscle strength, Physical performance, Frailty

## Abstract

**Purpose:**

Sufficient 25-hydroxyvitamin D (25(OH)D) concentrations might prevent a decline in physical performance, and are considered important for the prevention of frailty. This study investigates the association of serum 25(OH)D concentration with physical performance and frailty status in Dutch older adults.

**Methods:**

This cross-sectional study included 756 men and women, aged ≥ 65 years. Serum 25(OH)D concentration and frailty status (Fried criteria) were assessed in the total population. Screening for frailty status included functional tests of gait speed and hand grip strength. In a subgroup (*n* = 494), the Timed Up and Go test (TUG) and knee-extension strength were measured. Associations of serum 25(OH)D status with physical performance were examined by multiple linear regression. Prevalence ratios (PR) were used to quantify associations between serum 25(OH)D deficiency (< 50 nmol/L) and frailty.

**Results:**

In total, 45% of the participants were vitamin D deficient. Participants with vitamin D status < 50 and 50–75 nmol/L had significantly lower scores on the TUG and gait speed test, compared to participants with vitamin D status > 75 nmol/L. No significant associations with serum 25(OH)D concentrations were observed for handgrip strength or knee-extension strength. Participants with serum 25(OH)D status < 50 nmol/L were about two times more likely to be frail compared to participants with serum 25(OH)D status ≥ 50 nmol/L. No significant associations were observed between the pre-frail state and serum 25(OH)D status.

**Conclusion:**

In this study, serum 25(OH)D concentrations were significantly associated with frailty status and measures of physical performance, including gait speed and TUG, but not with strength-related outcomes.

## Introduction

Frailty is a geriatric syndrome associated with adverse health outcomes, such as physical disability, increased risk of falls, institutionalization, hospitalization and mortality [1]. To identify older people at risk, Fried et al. proposed a characterization of a frail state, using a clinical phenotype [2]. The definition consists of five physical components (weakness, slow walking speed, exhaustion, physical inactivity, and unintentional weight loss) and is now commonly applied in clinical research. The prevalence of frailty is relatively high among community-dwelling elderly, with 44% of seniors being pre-frail, and 10% being frail [3]. In view of the ageing population, the prevalence of the frailty syndrome will increase, which in turn will result in higher rates of hospitalization, and considerably burden the public health care costs [4]. As such, the need for interventions, supporting older people to remain healthy and independent, increases. One of the key features of frailty is profound muscle weakness and a decline in functional capabilities [2]. The cause of this loss in strength and function is multifactorial, and a low vitamin D status is suggested to be one of the risk factors [5, 6]. Vitamin D stimulates calcium absorption in the intestine and is responsible for the mineralization of bone and general functioning of cells throughout the body [7]. Deficient vitamin D concentrations [serum 25(OH)D < 50 nmol/L] [8] are common in frail older adults, with a prevalence reported up to 62% [9]. Low vitamin D concentrations have been associated with an impaired muscle function and an increased risk of being frail [9–11]. However, the strength and shape of these associations, and the ability to control for confounding factors differs between studies. Further characterization of the association between serum 25(OH)D concentration and frailty, but also the closely related functional parameters, might help to define consensus about the optimal vitamin D status for these health outcomes. Therefore, the aim of this study was to determine the association of serum 25(OH)D concentrations with physical performance and frailty status.

## Methods

### Study sample

In this study, we report data of 756 older adults that attended a screening visit for participation in the D-DOSE or D-FIT trial (clinicaltrial.gov registration: NCT01868945 or NCT02349282). These studies used similar recruitment strategies, inclusion criteria and measurement protocols, which allowed combining of datasets. Both studies were performed by the Division of Human Nutrition, Wageningen University, the Netherlands. Recruitment took place via university databases of volunteers and municipality registers of Wageningen and surroundings, inviting older adults ≥ 65 years to visit the study center to screen for eligibility to participate in one of the two trials. Details on eligibility criteria of the trials are described on clinicaltrial.gov. Participants were scheduled for the screening visit if they were 65 years or older and were interested to participate in the intervention study. In case people were not able to independently travel to the study center, a pick-up service was arranged. Visits took place between May 2013 and April 2015. During the visits, data on general characteristics, serum 25(OH)D status and frailty criteria (gait speed, handgrip strength, physical activity, weight loss and self-reported exhaustion) were collected in all participants. The screening protocol of the D-FIT trial (NCT02349282) contained additional measures of muscle strength and physical function to evaluate the willingness and ability of potential participants to comply with the study measures. As such, these additional measures are reported for a subgroup of 494 participants. Before screening, all participants signed informed consent and study protocols were approved by the ethical committee of Wageningen University.

### Serum 25-hydroxyvitamin D

Serum blood samples were collected during the screening visit and participants were instructed to remain fasted or take only a light meal. Samples were centrifuged, stored at − 80 °C and analyzed within one month after collection. Serum samples were analyzed using LC-MS/MS to measure total 25(OH)D concentration, which reflects the sum of serum 25(OH)D2 and 25(OH)D3. Samples collected for the D-DOSE study (*n* = 259) were analyzed at the Endocrine Laboratory of the VU University Medical Centre, Amsterdam, The Netherlands [12]. The intra-assay and inter-assay coefficients of variation were below 6 and 8%, respectively. Serum 25(OH)D samples collected for the D-FIT study (*n* = 497) were analyzed at the Department of Clinical Chemistry, Canisius Wilhelmina Hospital, Nijmegen, The Netherlands. The intra-assay and inter-assay coefficients of variation were below 4 and 7.5%, respectively [13]. Both laboratories are DEQAS-certified and the comparability of the LC-MS/MS methods between these two laboratories has been published previously, which indicated good agreement between methods [14].

### Physical performance

Handgrip strength (HGS) was measured on the dominant hand by taking the mean of three attempts (Jamar® hydraulic hand-held dynamometer, Patterson Medical, IL, USA). Mean gait speed was assessed by taking the average time, of two attempts, to walk a course of 15 feet. In a subgroup (*n* = 494), the Timed Up and Go test (TUG) and maximal isometric knee-extension strength were assessed. The TUG test is a test of functional ability to rise from a chair, walk 3 ms, make a turn, and walk back to the chair to sit down again. The average time to complete this test, out of two attempts, was recorded. Knee-extension strength was measured using the MicroFET hand-held dynamometer (Hoggan Health Inc., West Jordan, UT, USA). Participants were asked to sit upright with their knees in a 90° angle. Maximal strength (Newton) was measured three times per leg with 5 s of muscle contraction and 60-s of rest between repetitions. The average muscle strength of the right leg was used for analysis. All measurements were performed by examiners trained to regularly perform these tests according to study protocol and standardized verbal encouragement was provided.

### Fried frailty criteria

Frailty status was assessed using the criteria published by Fried et al. [2]. These consist of five criteria as described in Table 1: unintentional weight loss (in the past year, by questionnaire), self-reported exhaustion (CES-D questionnaire) [15], weakness (handgrip strength), slow walking speed (gait speed), and low physical activity levels (Short version of the Minnesota questionnaire) [16]. According to the frailty definition of Fried et al., a participant scores non-frail when no criteria are present, pre-frail when one or two criteria are present and frail when three or more criteria are present [2].

**Table 1:** Diagnosis of frailty according to the Fried criteria [1]

Criterion	Measured method	Cut-offs by sex and height			
Weight loss	Unintentional weight loss	Weight loss ≥ 4.5 kg in prior year			
Grip strength	Handgrip strength	Men		Women	
		BMI ≤ 24	≤ 29 kg	BMI ≤ 23	≤ 17 kg
		BMI 24.1–26	≤ 30 kg	BMI 23.1–26	≤ 17.3 kg
		BMI 26.1–28	≤ 30 kg	BMI 26.1–29	≤ 18 kg
		BMI > 28	≤ 32 kg	BMI > 29	≤ 21 kg
Physical activity	Short version of the Minnesota Questionnaire	Men		Women	
		≤ 1.6 MJ		≤ 1.1 MJ	
Gait speed	Gait speed 4.57-m course	Men		Women	
		Height ≤ 173 cm ≥ 7 s Height > 173 cm ≥ 6 s		Height ≤ 159 cm ≥ 7 s Height > 159 cm ≥ 6 s	
Self-reported exhaustion	CES-D Depression Scale	Participants scored positive if one of the following statements were present for ≥ 3d/wk: (a) I felt that everything I did was an effort; (b) I could not get going			

### Covariates

Questionnaires were used to record general participant characteristics such as, age, sex, ethnicity (Caucasian, other), physical activity (short version of the Minnesota questionnaire) [16], vitamin D supplement use, smoking status, alcohol intake, and the number of chronic diseases (including heart failure, hypertension, diabetes mellitus, renal insufficiency, liver disease or cancer). A stadiometer was used to measure the height of the participants, and a calibrated analog scale was used to measure their weight. BMI was calculated as kg/m^2^. In addition, laboratory site and season of blood collection (winter: December–February, spring: March–May, summer: June–August, autumn: September–November) were recorded.

### Statistical analyses

Characteristics of the study population are described as mean (SD), median (25th–75th percentile) or number (%) of categorical class. Serum 25(OH)D concentrations < 50 nmol/L are generally considered deficient [8, 17], and a status between 50 and 75 or > 75 nmol/L is suggested for optimal muscle health and physical performance [18, 19]. Serum 25(OH)D was categorized accordingly, with the latter (> 75 nmol/L) being the reference category. Differences between categories of serum 25(OH)D concentration were examined by one-way ANOVA for continuous variables, Kruskal–Wallis test in case of skewed variables and Chi-square tests for categorical variables. The association between serum 25(OH)D concentration and measures of physical performance (TUG and gait speed) and muscle strength (handgrip strength and knee-extension strength) were explored for nonlinearity by restricted cubic spline regression. As associations with TUG and hand grip strength tended to be nonlinear, all outcomes were further explored across categories of serum 25(OH)D. Multiple linear regression models were adjusted for factors known to be related to both serum 25(OH)D and physical performance. Model 1 was adjusted for age, sex and laboratory site. Model 2 was additionally adjusted for BMI and season of blood collection, and model 3 was additionally adjusted for ethnicity, physical activity, alcohol intake, smoking and number of diseases. A Cox Proportional Hazards analysis with robust error variance was performed to calculate prevalence ratios (PR) of participants being pre-frail or frail across categories of serum 25(OH)D status. By assigning a constant risk period to all participants, the obtained hazard ratio can be considered a PR [20]. Models including frailty as dependent variable were not corrected for physical activity, as this measure is also included in the definition of frailty status. Previous studies identified sex as a possible effect modifier in the association between vitamin D and physical performance [21]. Therefore, interaction terms including sex were added to the final models. A *P* value of ≤ 0.1 was considered significant to retain an interaction term in the model. All analyses were performed using statistical software package SAS version 9.2 (SAS Institute Inc., Cary, NC, USA) or using the R software package version 3.3.1 (R Foundation for Statistical Computing, Vienna, Austria). A two-sided *P* value of ≤ 0.05 was considered statistically significant.

## Results

Table 2 shows the general characteristics of the study population in total, and by categories of serum 25(OH)D status. The mean ± SD age of the study population was 74 ± 6 years and 55% were men. Mean BMI was 27.1 ± 3.5 kg/m^2^ and year-round median serum 25(OH)D status was 54 (38–72) nmol/L. Participants in the deficient serum 25(OH)D category (< 50 nmol/L) were more likely to be men and more likely to have a higher BMI compared to participants in the higher categories of serum 25(OH)D status. Season of blood collection was significantly different between the vitamin D categories, with 81% of the vitamin D deficient participants measured in the winter/ spring. Of all participants, 12% reported to use a vitamin D supplement. A significant difference was observed in the number of supplement users across categories, with 4% in the deficient category and 18 and 19% in the two higher categories. Most participants scored non-frail according to the Fried criteria, namely 57%, followed by 39% scoring pre-frail and 4% scoring frail.

**Table 2:** Participant characteristics

	Serum 25-hydroxyvitamin D				
	Total *n* = 756	< 50 nmol/L *n* = 340	50–75 nmol/L *n* = 254	> 75 nmol/L *n* = 162	*P* value
Men, *n* (%)	416 (55)	217 (64)	125 (49)	76 (46)	< 0.01
Age, year	73.8 ± 6.4	74.1 ± 6.6	74.0 ± 6.2	72.9 ± 5.9	0.08
BMI, kg/m^2^	27.1 ± 3.5	27.5 ± 3.7	27.0 ± 3.3	26.2 ± 3.1	< 0.01
Caucasian, *n* (%)	736 (98)	327 (97)	250 (98)	159 (98)	0.49
Independent living, *n* (%)^a,h^	723 (96)	320 (95)	243 (96)	160 (99)	0.10
Non-smokers, *n* (%)^a^	705 (94)	311 (92)	239 (94)	155 (96)	0.31
Alcohol consumers, *n* (%)^a^	598 (79)	269 (80)	197 (78)	132 (82)	0.61
25(OH)D, nmol/L	54 (38–72)	36 (29–42)	62 (58–67)	91 (84–100)	< 0.01
Calcium	2.24 (0.09)	2.24 (0.09)	2.25 (0.10)	2.24 (0.10)	0.43
VitD suppl. users, *n* (%)^b^	88 (12)	13 (4)	45 (18)	30 (19)	< 0.01
Season, *n* (%)^i^					
Summer–autumn	259 (34)	64 (19)	101 (40)	94 (58)	< 0.01
Winter–spring	497 (66)	276 (81)	153 (60)	68 (42)	
Number of diseases, *n* (%)^c^					
0	380 (51)	159 (47)	130 (52)	91 (56)	0.36
1–2	353 (47)	167 (50)	117 (46)	69 (43)	
≥ 3	17 (2)	10 (3)	5 (2)	2 (1)	
Physical activity, MJ/week^d^	8.4 (4.5–13.0)	7.8 (4.5–13.1)	8.5 (4.3–12.0)	9.6 (5.4–15.1)	0.11
TUG, s^j,d^	9.8 ± 2.4	9.9 ± 2.5	9.9 ± 2.3	9.1 ± 2.2	0.04
Gait, m/s^e^	1.06 ± 0.20	1.05 ± 0.21	1.05 ± 0.19	1.10 ± 0.20	0.03
Knee-extension, N^j^	328 ± 104	336 ± 103	324 ± 104	301 ± 106	0.04
HGS, kg^f^	28.9 ± 9.5	29.4 ± 9.3	28.1 ± 9.6	28.9 ± 9.7	0.23
Frailty, *n* (%)^g^					
Non-frail	425 (57)	183 (55)	142 (57)	100 (62)	0.16
Pre-frail	289 (39)	131 (39)	98 (39)	60 (37)	
Frail	33 (4)	20 (6)	11 (4)	2 (1)	
Positive on frailty					
Weight loss, *n* (%)	23 (3)	14 (4)	5 (2)	4 (3)	0.28
Exhaustion, *n* (%)^a^	115 (15)	51 (15)	41 (16)	23 (14)	0.86
Physical activity, *n* (%)^a^	54 (7)	26 (8)	20 (8)	8 (5)	0.46
HGS, *n* (%)^a^	213 (28)	102 (30)	76 (30)	35 (22)	0.10
Gait, *n* (%)^c^	49 (7)	26 (8)	16 (6)	7 (4)	0.34

Values presented are mean ± SD or median (25th–75th percentile). Between-group differences explored by one-way ANOVA, Kruskal–Wallis test or Chi-square test

*25(OH)D* 25-hydroxyvitamin D, *BMI* Body Mass Index, *VitD suppl. users* vitamin D supplement users; *TUG* Timed Up and Go, *HGS* Hand grip strength, *N* Newton

^a^3 missing values

^b^5 missing values

^c^6 missing values

^d^1 missing value

^e^7 missing values

^f^4 missing values

^g^9 missing values

^h^Assisted living includes: home care or service flat

^i^Winter–spring: Dec–May, summer–autumn: Jun–Nov

^j^Subgroup *n* = 494

Table 3 shows the association between serum 25(OH)D concentration and measures of physical performance. There was an inverse association between serum 25(OH)D and TUG test scores, which remained significant after full adjustment for confounders. Compared with the reference category (> 75 nmol/L), participants with serum 25(OH)D concentrations < 50 nmol/L (*β* 0.73, 95% CI 0.14; 1.32) and 50–75 nmol/L (*β* 0.83, 95% CI 0.21; 1.45) had significantly higher TUG scores, indicating more time needed to complete the test. Likewise, participants with serum 25(OH)D status < 50 nmol/L (*β* − 0.04, 95% CI − 0.08; − 0.01) and status between 50 and 75 nmol/L (*β* − 0.04, 95% CI − 0.07; − 0.01) had significantly lower gait speed scores, compared with the reference category. Serum 25(OH)D categories were not associated with handgrip strength and knee-extension strength. The effect of vitamin D supplement use was explored by adjusting for this variable in the final model; however, this did not change the interpretation of results. Furthermore, interaction analyses did not suggest significant modification of the associations by sex.

**Table 3:** Association between serum 25-hydroxyvitamin D status and physical performance

	Serum 25-hydroxyvitamin D			*n*
	< 50 nmol/L	50–75 nmol/L	> 75 nmol/L	
	*β* (95% CI)	*β* (95% CI)	Reference group	
TUG, s				
Model 1	0.85 (0.24; 1.45)**	0.83 (0.19; 1.47)*	0 (ref)	493
Model 2	0.77 (0.18; 1.36)*	0.84 (0.22; 1.47)**	0 (ref)	493
Model 3	0.73 (0.14; 1.32)*	0.83 (0.21; 1.45)**	0 (ref)	488
Gait, m/s				
Model 1	− 0.06 (− 0.10; − 0.02)**	− 0.05 (− 0.09; − 0.01)**	0 (ref)	749
Model 2	− 0.05 (− 0.09; − 0.01)**	− 0.04 (− 0.08; − 0.01)*	0 (ref)	749
Model 3	− 0.04 (− 0.08; − 0.01)*	− 0.04 (− 0.07; − 0.01)*	0 (ref)	745
HGS, kg				
Model 1	− 0.93 (− 2.25; 0.38)	− 0.71 (− 2.02; 0.61)	0 (ref)	752
Model 2	− 1.06 (− 2.38; 0.26)	− 0.84 (− 2.15; 0.47)	0 (ref)	752
Model 3	− 0.92 (− 2.25; 0.40)	− 0.78 (− 2.10; 0.53)	0 (ref)	748
Knee-extension, N				
Model 1	7.74 (− 15.03; 30.50)	12.23 (− 11.95; 36.42)	0 (ref)	494
Model 2	7.70 (− 15.10; 30.50)	13.09 (− 11.11; 37.29)	0 (ref)	494
Model 3	9.89 (− 12.82; 32.60)	12.71 (− 11.37; 36.80)	0 (ref)	489

Analyzed by multiple linear regression

*Model 1* adjusted for age, sex and laboratory site, *Model 2* adjusted for age, sex, laboratory site, BMI and season, *Model 3* adjusted for age, sex, laboratory site, BMI, season, ethnicity, physical activity, alcohol, smoking and number of diseases, *TUG* Timed Up and Go, *HGS* Hand grip strength, *N* Newton

*P < 0.05

**P < 0.01

Table 4 shows the association between serum 25(OH)D concentrations and frailty status. As only two participants scored frail in the > 75 nmol/L category, the 50–75 and > 75 nmol/L categories were combined to further explore the association between serum 25(OH)D and frailty status. Participants with serum 25(OH)D status < 50 nmol/L were about two times more likely to be frail (PR = 2.30, 95% CI 1.11; 4.76, *P* = 0.02), compared to participants with serum 25(OH)D status ≥ 50 nmol/L. The effect of vitamin D supplement use was explored by adjusting for this variable in the final model. This attenuated the prevalence ratio, but the association remained significant (PR = 2.16, 95% CI 1.04; 4.52, *P* = 0.04). When comparing non-frail vs. pre-frail older adults (or pre-frail and frail combined), no significant associations were observed with serum 25(OH)D status.

**Table 4:** Prevalence ratios (PR) and 95% CIs for frailty status of participants with serum 25-hydroxyvitamin D concentrations < 50 versus ≥ 50 nmol/L

	Serum 25-hydroxyvitamin D		*n*
	< 50 nmol/L	≥ 50 nmol/L	
	PR (95% CI)	Reference group	
Frail vs. non-frail			
Model 1	2.24 (1.06; 4.75)*	1 (ref)	458
Model 2	2.07 (1.02; 4.20)*	1 (ref)	458
Model 3	2.30 (1.11; 4.76)*	1 (ref)	453
Pre-frail vs. non-frail			
Model 1	1.10 (0.91; 1.32)	1 (ref)	714
Model 2	1.08 (0.90; 1.29)	1 (ref)	714
Model 3	1.06 (0.88; 1.26)	1 (ref)	711
Pre-frail or frail vs. non-frail			
Model 1	1.14 (0.97; 1.35)	1 (ref)	747
Model 2	1.13 (0.96; 1.32)	1 (ref)	747
Model 3	1.10 (0.93; 1.29)	1 (ref)	742

Analyzed by Cox Proportional Hazards analysis

*Model 1* adjusted for age, sex and laboratory site, *Model 2* adjusted for age, sex, laboratory site, BMI and season, *Model 3* adjusted for age, sex, laboratory site, BMI, season, ethnicity, alcohol, smoking and number of diseases. Models are not corrected for physical activity, as this measure is also included in frailty status

*P < 0.05

## Discussion

In this study, serum 25(OH)D concentrations were significantly associated with physical performance and frailty status in a population of community-dwelling older adults. To date, no generally accepted recommendation on the optimal serum 25(OH)D status for muscle function is present, with the IOM proposing concentrations of 30–50 nmol/L for older adults [8], and others supporting thresholds of 75 nmol/L or higher [18, 19]. Our results indicated that serum 25(OH)D status < 50 nmol/L, but also between 50 and 75 nmol/L, were associated with lower functioning on TUG and gait speed tests, when compared to serum 25(OH)D status > 75 nmol/L. Similar associations were observed in two large cohorts, where low serum 25(OH)D was associated with physical performance, and the strongest associations were observed on walking tests [22, 23]. In addition, comparable effect estimates were reported in a study of older adults at risk of disability, with slower walking speed (mean difference 0.04 m/s) in vitamin D deficient older adults (< 50 nmol/L) compared to those with a sufficient status [24]. The association with TUG was also observed in previous studies, where higher vitamin D concentrations were associated with a faster performance on the TUG test [21, 25]. In a study by van Dam et al., women with serum 25(OH)D concentrations ≤ 80 nmol/L showed lower TUG scores (mean difference 0.77 s) than those who had a serum 25(OH)D status ≥ 115 nmol/L, and the lower 25(OH)D category appeared predictive of a greater decline in function over a period of 2.5 years [21]. In addition, several studies report an association between vitamin D deficiency and reduced handgrip strength [23, 26], or leg extension strength [27, 28], albeit not all [29]. In our study, no significant association was observed between serum 25(OH)D concentration and measures of muscle strength. Mechanistically, the link between vitamin D and muscle function is explained via the regulation of calcium and phosphate, necessary for muscle contraction, or via the activation of the vitamin D receptor (VDR) in muscle cells [30]. However, the presence of the VDR is also observed in neurons and glial cells in several regions of the brain, which suggests a role of vitamin D in the neuromuscular system [31, 32]. Vitamin D deficiency is associated with an increased postural sway and greater risk of falling [33]. Moreover, a previous trial showed that vitamin D supplementation improved balance with 9% in vitamin D deficient older adults [34]. It is thus plausible that vitamin D status is more strongly associated with complex functional parameters rather than muscle strength due to its suggested role in neurological processes of motor performance. However, more research is needed in this field.

Vitamin D deficient participants (< 50 nmol/L) were ~ 2 times as likely to score frail, compared to those with sufficient serum 25(OH)D concentrations. This is in line with most previous studies investigating this association [9, 35, 36]. In the Longitudinal Aging Study Amsterdam (LASA), participants with 25(OH)D status between 25 and 50 nmol/L were 1.7 times as likely to be frail, and those with serum status below 25 nmol/L were 2.6 times as likely to be frail compared to the reference group with vitamin D status > 50 nmol/L [37]. Likewise, in the NHANES III study, older adults with vitamin D deficiency (< 37 nmol/L) were 3.7 times as likely to score frail on the Fried criteria compared to the reference group (≥ 75 nmol/L) [11]. Also, pre-frail individuals are regarded as a suitable target group for intervention studies to prevent further functional decline [38]. In our study, serum 25(OH)D concentrations were not associated with the pre-frail state. While the prevalence of pre-frailty was higher than frailty, the non-significant findings might relate to the fact that the pre-frail state is a less distinct condition as only one or two Fried criterion need to be present to score positive on pre-frailty. Nevertheless, a prospective study reported that serum 25(OH)D status ≥ 50 nmol/L tended to prevent a decline from the pre-frail to frail state over a period of 3 to 6 years [39], which might indicate that prevention of vitamin D deficiency is also relevant in this stage. Furthermore, sex has been reported as an effect modifier in the association between 25(OH)D and frailty [40]. In our study, the prevalence of frailty was relatively low (4%), limiting reliable testing of effect modification across such a small sample of cases.

Overall, the association between vitamin D status and frailty appears to be consistent, with lower serum 25(OH)D status associated with an increased likelihood of being frail. However, the causality of the association remains to be determined, given the cross-sectional design of these reported associations. An important determinant of vitamin D status is sun exposure, which closely relates to being outdoors and physically active. As frail older adults might stay more indoors, reverse causation is plausible. Autier et al. recently discussed that the serum 25(OH)D status might not be a cause of adverse health outcomes, but a marker of ill health [41]. While we controlled for a broad range of lifestyle and health-related factors, correcting for confounding remains challenging. As we used questionnaires to estimate physical activity status and season to correct for sun exposure, residual confounding cannot be excluded. Besides the factors inherent to the cross-sectional design of this study, other limitations should be noted. The prevalence of frailty was relatively low when compared to the reported prevalence of 10% for physical frailty in community-dwelling older adults [3]. The low prevalence might relate to the fact that this population consists of older adults that were willing to participate in an intervention trial and had likely a better health status or were more mobile compared to the general population, limiting the generalizability of the study findings. Also, parathyroid hormone (PTH), could potentially mediate the association between serum 25(OH)D status and physical performance or frailty, but was not measured in this study. Nevertheless, Pabst et al. investigated the mediating effect of PTH on frailty, but the attenuation of OR was small, suggesting an independent association with 25(OH)D [36]. Strengths of this study include the broad range of vitamin D concentrations measured in this population, the relatively high prevalence of vitamin D deficiency, and the fact that, besides the measurement of frailty, we included measures of lower extremity strength and TUG to reflect overall body function and strength.

With only 12% of the participants using a vitamin D supplement, 45% of our study population was vitamin D deficient. Identifying older adults at risk of vitamin D deficiency might be important given the possible predisposed risk of frailty. In this study, associations were observed between 25(OH)D status and the performance on the TUG and gait speed test. Both tests represent the ability of motor performance and balance control, supporting the plausible modulatory role of vitamin D in fall prevention [42]. Although the observed associations represent only small clinically meaningful changes [43, 44], if causal, these findings might be relevant for public health.
